# Dysfunctions of sleep and the circadian rhythm in Huntington’s disease

**DOI:** 10.3389/fneur.2025.1693307

**Published:** 2025-11-07

**Authors:** Paulina Kaczmarska, Monika Rudzińska-Bar

**Affiliations:** Department of Neurology, Medical College, Andrzej Frycz Modrzewski Krakow University, Krakow, Poland

**Keywords:** Huntington’s disease, sleep, sleepiness, insomnia, circadian rhythm, neurodegeneration, cognitive impairment, quality of life

## Abstract

Sleep and circadian rhythm disturbances are highly prevalent in Huntington’s disease (HD) and increasingly well characterized through both subjective assessments—such as questionnaires—and objective techniques, including polysomnography and actigraphy. These disturbances encompass a broad range of features, including sleep fragmentation, altered sleep architecture, insomnia, and delayed circadian phase, often emerging years before motor onset. This review outlines the clinical relevance of sleep dysfunction in HD and its potential bidirectional relationship with neurodegeneration, highlighting mechanisms that may contribute to symptom progression and disease burden. Emerging evidence, primarily from preclinical HD models, suggests that dysregulated sleep–wake and circadian processes may accelerate underlying neuropathological changes. We describe the clinical presentation of sleep-related disturbances in HD, including abnormal nocturnal motor activity and insomnia, while also noting less frequent phenomena such as sleep-related breathing disturbances and REM sleep behavior disorder (RBD). In parallel, we summarize findings on circadian misalignment and its association with altered melatonin signaling, hypothalamic pathology, and disrupted gene expression rhythms. Sleep and circadian disturbances are further linked to cognitive impairment and reduced quality of life. Finally, the review outlines current therapeutic strategies—derived from studies in other neurodegenerative disorders, animal models, and the first randomized controlled trial conducted in HD—highlighting the need for targeted interventions. Altogether, the available evidence suggests that sleep and circadian dysfunctions are modifiable components of HD that warrant greater attention in both research and clinical practice.

## Introduction

Huntington’s disease (HD) is a progressive neurodegenerative disorder caused by an autosomal dominant inheritance of an expanded CAG repeat in exon 1 of the huntingtin (HTT) gene. This genetic mutation leads to toxic protein accumulation in neurons. It is characterized by a triad of motor, cognitive, and psychiatric symptoms, typically emerging between the ages of 35 and 50. The number of CAG repeats is inversely correlated with the age of symptom onset. Neurodegenerative changes primarily affect the basal ganglia, with atrophy reaching up to 70% of the volume of the caudate nucleus, globus pallidus, and putamen (1). However, they are not confined to these structures.

The significance of sleep disorders and circadian rhythm disturbances are often under-recognized features of HD. Emerging evidence suggests that these abnormalities may not only accompany the disease but also precede the clinical onset of symptoms, contributing to cognitive decline and psychiatric disturbances. While motor symptoms such as chorea typically diminish during sleep, disruptions in sleep architecture and regulation of the circadian rhythm extend beyond movement abnormalities, involving complex neurodegenerative processes.

This review aims to provide an overview of the current understanding of sleep and circadian rhythm disturbances in HD, explore the underlying neuropathological mechanisms, and discuss potential therapeutic strategies to improve sleep quality and overall disease management.

### Sleep and neurodegeneration

Sleep disorders and circadian rhythm disturbances are not traditionally considered core symptoms of HD, but there is growing awareness of their role in the natural course of the disease. In other neurodegenerative disorders, particularly Alzheimer’s disease (AD) and Parkinson’s disease (PD), sleep-related symptoms have been widely recognized and have been firmly established as part of the clinical spectrum of these conditions (2, 3).

The relationship between sleep disturbances and neurodegenerative conditions remains incompletely understood. Researchers emphasize its potential bidirectional nature (4). On the one hand, progressive degenerative damage to structures involved in sleep and circadian rhythm regulation, such as the brainstem and hypothalamus, leads to sleep disturbances. This, in turn, significantly impairs the patient’s quality of life. On the other hand, sleep abnormalities and circadian disruptions may exacerbate the neurodegenerative process.

In healthy individuals, even short-term sleep deprivation, such as shift work or jet lag, leads to transient cognitive impairments, including reduced attention, memory deficits, and diminished executive function. It also contributes to greater emotional instability, impulsivity, and increased aggression (5–7). Given the severe neurological burden in conditions like HD, the negative impact of sleep disturbances is likely to be even more pronounced. Evidence from animal studies suggests a direct link between sleep disturbances and neurodegeneration. First, sleep disruption promotes central nervous system neuroinflammation (8). Second, it impairs synaptic modulation, particularly in the hippocampus, a structure critical for memory consolidation (9).

Another proposed mechanism of neurodegeneration involves dysfunction of the glymphatic system. This system, composed of perivascular spaces and astrocytes expressing aquaporin-4, is responsible for clearing toxic proteins from the brain’s interstitial space, including beta-amyloid and tau protein (10–12). Its activity is regulated by sleep and the circadian rhythm. Disruptions in sleep, particularly in slow-wave sleep (SWS) when glymphatic clearance is most active, may contribute to neurodegeneration by promoting the accumulation of pathological proteins (13).

### Clinical presentation of sleep disturbances in HD

Although sleep disturbances are not considered main symptoms of HD, they are often overlooked during the diagnostic process. Patients rarely report such issues, which may result from a lack of insight into their condition and the tendency to downplay their significance. However, when asked directly, as many as 88% of symptomatic HD patients acknowledge experiencing subjective sleep difficulties (14).

Studies using the Pittsburgh Sleep Quality Index (PSQI) have indicated a higher prevalence of sleep disturbances in HD patients compared to healthy controls (58.1% vs. 34.9%) (15). Additionally, a questionnaire designed for this population by researchers at the University of Cambridge revealed that 60% of HD patients consider sleep problems a significant aspect of their health status (16).

Recent large-scale data from the Huntington’s Disease Burden of Illness (HDBOI) study (17) further emphasize the clinical relevance of these symptoms. Among 2,094 individuals with manifest HD, 482 provided answers to sleep-related items. An overwhelming 91% of respondents reported experiencing sleep problems. The prevalence of these disturbances increased significantly with disease progression, exhibiting a statistically significant trend across early, mid, and advanced clinical stages (*p* < 0.05). Although stage-specific percentages were not disclosed, these findings are consistent with prior reports indicating that sleep dysfunction tends to worsen as the disease advances (4, 18).

Despite the high prevalence of self-reported sleep problems in HD, subjective assessments show poor correlation with polysomnographic findings. This discrepancy encompasses both over- and under-estimation of various sleep parameters (19).

### Objective assessment of sleep disturbances in HD

Studies employing objective methods, allowing for definitive confirmation of sleep pathology through polysomnography (PSG) recordings, indicate that sleep disturbances emerge as early as the pre-symptomatic stage in carriers of the HD-related mutation (20, 21).

The electrophysiological pattern of sleep, characterized by brain activity recorded via electroencephalography (EEG), identifies two main phases: non-rapid eye movement (NREM) sleep and rapid eye movement (REM) sleep. During NREM sleep, sleep progressively deepens, and is divided into stages N1 and N2 (referred to as “light sleep”) and stages N3 and N4 (known as “deep sleep” or “slow-wave sleep”). The complete transition through all NREM stages takes approximately 60–90 min, followed by the REM phase. Initially lasting around 10 min, REM sleep precedes the return to NREM sleep, forming a cycle that repeats four to five times per night in healthy individuals (22). The NREM phase is longer and more profound in the early hours of sleep. As the night progresses, its intensity gradually decreases, while REM sleep occupies an increasing proportion of subsequent cycles (23). Distinct but interconnected brain centers regulate both sleep and wakefulness. Key structures involved in this process include the hypothalamus, thalamus, and tegmentum, as well as the orexin system, which facilitates dynamic transitions between sleep and wakefulness (24).

PSG provides a detailed analysis of sleep phases. It is a comprehensive diagnostic method that integrates simultaneous EEG, electromyography (EMG), and electrooculography recordings, offering precise insights into sleep architecture.

Polysomnographic studies in patients with HD indicate considerable variability in reported sleep structure abnormalities. To date, no pathognomonic sleep pattern for HD has been identified and consistently confirmed across studies. These discrepancies may stem from several factors, including the absence of genetic diagnostics in earlier research (the IT15/HTT gene was identified in 1993), differences in disease stages among participants, the influence of medications, and variations in study protocols (21, 25–28).

In 2019, a meta-analysis included seven polysomnographic studies with appropriately matched control groups. In three of these studies, polysomnographic recordings were performed over two nights, with data analysis limited to the second night. In the remaining four studies, recordings were restricted to a single night, omitting the so-called adaptation night. A total of 152 patients with genetically confirmed HD and 144 individuals from the control group were evaluated. In the HD group, characteristic patterns of sleep architecture disturbances were observed, including reduced sleep efficiency, increased time spent in light sleep (N1), and a shortened SWS and REM sleep duration. Additionally, significant sleep fragmentation was noted, reflected by prolonged wake after sleep onset (WASO) and increased REM sleep latency. These two parameters and the reduced duration of deep sleep significantly correlated with patient age in the HD group (29).

Subsequent video-PSG study analyzed data from 23 HD patients and 13 controls. The findings were consistent with previous research and were statistically confirmed. HD patients exhibited reduced sleep efficiency, increased WASO, and shortened durations of REM sleep. An interesting observation was the significantly higher number of transitions between sleep stages in the HD group compared to the control group (30).

Studies conducted in pre-symptomatic and early-manifest HD patients suggest that sleep disturbances may precede the clinical onset of the disease by many years and worsen as the disease progresses (21, 26). In this patient group, two particularly notable features were REM sleep shortening and significant sleep fragmentation (20, 31).

A recent 12-year longitudinal study by Voysey et al. provided the first prospective, polysomnography-based characterization of sleep abnormalities in Huntington’s disease. HD gene carriers in premanifest and early manifest stages underwent repeated in-laboratory polysomnographic assessments at defined intervals, supplemented by long-term actigraphy.

Two principal abnormalities were identified: sleep stage instability, reflected by frequent transitions between sleep stages, and sleep maintenance insomnia, characterized by WASO. Sleep stage instability emerged early in the disease trajectory and progressed gradually, whereas sleep maintenance insomnia became more pronounced closer to clinical onset (phenoconversion). Notably, baseline measurements of sleep stage instability predicted phenoconversion within the 12-year follow-up period (AUC = 0.81), underscoring its potential value as an early biomarker. Elevated WASO also correlated with increased serum neurofilament light chain (NfL) levels, a marker of neurodegeneration (32).

The disrupted sleep architecture patterns and their progression with disease advancement are features shared by HD and other neurodegenerative disorders, particularly AD and PD (33). This may serve as further evidence of a significant relationship between sleep disturbances and neurodegeneration.

### HD and periodic limb movement disorder

From a clinical perspective, two main types of nocturnal sleep disturbances have been identified in HD patients. The first is abnormal motor activity during sleep, though its characteristics remain unclear. In individual studies, such movements have been reported as chorea or repetitive ballistic movements (34), which is noteworthy, as these findings contradict classical reports suggesting that chorea tends to diminish during sleep and, in some cases, may even disappear entirely (35). Moreover, several investigations have identified periodic limb movement disorder (PLMD), characterized by repetitive, stereotyped movements predominantly involving the lower extremities (21, 36).

A video-PSG study involving 30 HD patients found PLMs in all participants. These movements were present across all sleep stages, although their intensity was lower during REM sleep. In some cases, upper limb involvement was also observed (25).

However, these findings contradict another video-PSG study involving 29 HD patients. In this study, significant motor agitation during sleep was observed. These movements included position changes, pulling on pillows or sheets (with noticeably greater clumsiness than in the control group), aggressive kicking, extensor posturing resembling opisthotonus, bruxism, and vocalizations. These disturbances occurred during physiological arousal during the transition between NREM and REM sleep. However, they lacked regularity, ruling out their PLM classification (28).

Building on these earlier observations, a 2025 study aimed to clarify whether abnormal nocturnal movements in early HD occur during sleep itself or arise following brief awakenings. By linking long-term actigraphy data with later PSG findings, the authors demonstrated that elevated nocturnal motor activity was associated with increased wakefulness rather than movements within sleep, indicating these motor activities predominantly occur during periods of nocturnal wakefulness (32).

### HD and REM sleep behavior disorder

Given the increased number of arousals and prolonged duration of light sleep—both promoting heightened motor activity compared to deep sleep—it can be assumed that abnormal motor activity may persist significantly throughout the night, particularly in the advanced stages of the disease.

Unlike other neurodegenerative diseases, such as PD, dementia with Lewy bodies (LBD), and multiple system atrophy (MSA), HD is not primarily driven by dopaminergic dysfunction. As a result, REM sleep-related motor disturbances are not a characteristic feature of its clinical presentation. Based on clinical interviews, 23% of patients and their caregivers report symptoms suggestive of REM sleep behavior disorder (RBD) (37). Nevertheless, polysomnographic assessments have confirmed the presence of RBDs in only 12% of HD patients (19). Moreover, another study utilizing PSG did not identify any cases of RBD (25).

### HD and insomnia

Another sleep disorder observed in HD patients is insomnia, identified in up to two-thirds of individuals when formal clinical criteria are applied—namely, difficulty initiating or maintaining sleep, accompanied by daytime consequences, occurring at least four nights per week for a minimum duration of three months (21). Questionnaire-based studies comparing HD patients to matched controls have demonstrated significantly delayed sleep onset, longer total sleep duration, greater use of hypnotic agents, and increased daytime dysfunction among affected individuals (15). Despite preserved total sleep time, sleep in HD is markedly less consolidated, with increased fragmentation and frequent nocturnal awakenings. As discussed earlier, sleep maintenance insomnia—defined as difficulty staying asleep—emerges as a salient feature near the transition from premanifest to manifest HD. Its severity correlates strongly with impairments in attention, executive functioning, and psychomotor speed, independent of disease stage and other confounding factors. These findings support the interpretation of sleep maintenance insomnia not only as a clinically meaningful symptom but also as a promising therapeutic target for improving quality of life and potentially modifying disease progression in HD (32).

Findings regarding excessive daytime sleepiness remain inconclusive. Some studies suggest no significant difference compared to control groups (15, 21, 38), while others indicate its presence in up to 30% of patients (39). These discrepancies may be due to the influence of coexisting depressive disorders, which have a statistically significant correlation with excessive daytime sleepiness (37).

### HD and obstructive sleep apnea

It is worth noting that, unlike other neurodegenerative disorders, sleep-related breathing disturbances are not typically observed in HD patients. Although minor alterations in respiratory patterns have been reported in some studies, their clinical relevance remains unclear (21, 40, 41). Notably, a 2024 meta-analysis demonstrated that individuals with Huntington’s disease dementia exhibit a markedly lower prevalence of sleep-related breathing disorders (16%) compared to those with Alzheimer’s disease dementia (89%) or Parkinson’s disease–related dementia (56%), indicating that such disorders are four to six times less common in HD than in other dementias (42). Overall, the available data suggest that OSA is not a prominent feature of sleep pathology in HD.

### Circadian rhythm disorders in HD

A study assessing 84 carriers of the mutant HD gene used detailed interviews and sleep assessment scales to evaluate sleep patterns. The results showed a significantly higher prevalence of delayed sleep onset and later wake times in HD carriers compared to a control group. These findings indicate the presence of a delay in the sleep phases of HD patients (15). This phenomenon has been linked to a delayed evening peak in melatonin secretion by the pineal gland, observed in individuals with early-stage HD (43).

In a study of 27 patients with the HD mutation and a control group, 24-h serum melatonin level monitoring was performed with measurements taken at hourly intervals. While a clear phase shift in melatonin secretion was not confirmed, findings indicated a reduction in average melatonin levels, decreased peak phase (acrophase) values, and diminished secretion amplitude. As the disease progressed, melatonin levels continued to decline. In patients at stage II/III, lower concentrations and reduced pulsatility of melatonin secretion were observed compared to the control group. Additionally, individuals in the pre-symptomatic phase of HD exhibited decreased melatonin levels, although this difference did not reach statistical significance (44). The Actiwatch-Neurologica system was used to assess locomotor activity, indicating circadian rhythm disturbances, reflected in an abnormal day-to-night activity ratio. However, these findings did not provide conclusive evidence of a sleep phase shift (45).

Actigraphy is a motion-based monitoring method that utilizes a wrist-worn device, allowing continuous recording of activity over extended periods. While there is considerable discrepancy between actigraphy and EEG in detecting nocturnal wakefulness—reflecting its limited precision in assessing detailed sleep architecture (46)—it remains a valuable and objective tool for evaluating overall 24-h activity patterns across different stages of HD.

In pre-HD, greater sleep fragmentation was observed in participants estimated to be within 15 years of clinical diagnosis compared with both healthy controls and those more distant from onset, despite comparable sleep efficiency. Intra-daily variability, reflecting day–night activity transitions, was lower, indicating preserved overall rest–activity rhythms. These findings suggest that sleep fragmentation represents an early and distinct feature of premanifest HD (47).

In a recent study involving 20 patients with early-mid manifest HD and matched healthy controls, seven-day continuous actigraphic monitoring revealed specific temporal peaks of motor activity. These occurred between 2:15 a.m. and 4:00 a.m.—a period typically dominated by deep NREM (N3) sleep in healthy individuals—as well as around bedtime (approximately 40 min before to 120 min after sleep onset) and around awakening (20 min before to 50 min after get-up time). This distinct temporal activity pattern appears to reflect a disease-specific disturbance in circadian and may serve as a potential actigraphic biomarker of circadian dysregulation; however, more data from PSG studies and biochemical measures are required. If confirmed, such an approach could provide a practical, accessible, and patient-friendly tool for assessing sleep–wake disturbances in both clinical and research contexts (38).

The phenotypical assessment of circadian rhythm disturbances in HD remains particularly challenging due to the difficulty in distinguishing true circadian dysregulation from secondary sleep abnormalities. For instance, delayed sleep onset in HD patients may not necessarily reflect a genuine phase delay, but rather sleep initiation insomnia—difficulty falling asleep despite expected biological timing. Conversely, prolonged morning sleep may arise from compensatory rest following fragmented or nonrestorative sleep, making it difficult to separate the two processes.

Additional insights into circadian rhythm disturbances in HD come from studies on animal models. In transgenic R6/2 mice, a progressive increase in daytime activity and a reduction in nighttime activity were observed. These changes worsened as the disease advanced, leading to a complete disruption of the circadian rhythm (48).

In all mammals, the SCN, located in the anterior hypothalamus, serves as the master regulator of circadian rhythms. Studies on animal models with HD have demonstrated pathological alterations in the neuronal activity rhythms of this region (48, 49). Additionally, biochemical disturbances have been identified, including reduced vasopressin secretion and vasoactive intestinal peptide levels within the SCN (50).

The activity of the central biological clock and peripheral “clocks” in non-neuronal tissues, as well as the circadian rhythm of each nucleated cell, depends on the expression of so-called “clock genes.” A transcription-translation feedback loop mechanism regulates their cyclic activation.

Core clock genes, such as *Period* (PER) and *Cryptochrome* (CRY), are activated at the beginning of the circadian cycle by CLOCK/BMAL protein heterodimers. PER and CRY protein complexes gradually accumulate in the cell nucleus, inhibiting CLOCK/BMAL activity and closing the feedback loop. During the night, PER/CRY complexes undergo degradation, allowing the next cycle to begin. This process takes approximately 24 h, generating the circadian rhythm (24, 51, 52).

The activity of the SCN is regulated by external time cues, known as Zeitgebers (German for “time givers”), including light and physical activity. Its primary function is to coordinate the synchronization of peripheral biological clocks with each other and the solar cycle. This process occurs through neuronal pathways, such as the autonomic nervous system and humoral mechanisms, in which melatonin and cortisol play key roles (52).

Studies on animal models with HD have unequivocally confirmed abnormal expression of clock genes (45, 48). Interestingly, experiments using isolated SCN cells from transgenic R6/2 mice exhibited severe circadian rhythm disturbances but revealed that clock gene expression remained intact under *in vitro* conditions. Furthermore, the cyclic activity of these genes was preserved, suggesting that the observed disruptions in circadian rhythms may result from impaired regulatory mechanisms rather than intrinsic defects in clock gene function. Notably, interventions such as alprazolam administration (53) or a regular feeding schedule (54) improved circadian rhythms in these animals.

These findings suggest that HTT’s direct impact on the cellular circadian clock is limited. The observed disruptions in clock gene expression, which contribute to circadian rhythm abnormalities, may instead result from hormonal factors (e.g., melatonin and cortisol levels) or behavioral influences (4).

Studies on transgenic sheep carrying the HD mutation (OVT73) further support the significance of external factors. As these animals aged, they exhibited progressively worsening circadian rhythm disturbances, mirroring clinical observations in humans. These abnormalities were most pronounced in groups composed solely of HD-mutant sheep. Interestingly, sheep housed in mixed herds, where healthy individuals were also present, displayed less severe circadian disruptions. This suggests that social interactions may help mitigate specific behavioral symptoms associated with HD (55).

Hypothalamic neurodegeneration in HD affects orexinergic neurons in the lateral hypothalamus (56, 57). Orexins, which physiologically stabilize wakefulness, are crucial in regulating the sleep–wake cycle. However, studies of cerebrospinal fluid (CSF) in HD patients have not revealed alterations in orexin levels (58). Magnetic resonance imaging (MRI) has shown a weak correlation between hypothalamic degeneration and clinically observed disturbances in sleep and circadian rhythms (59).

The neurodegenerative process in HD also affects other brain regions that may directly or indirectly influence sleep and circadian rhythm. Brainstem atrophy, which houses a key structure responsible for regulating transitions between NREM and REM sleep, has been well documented (60). Additionally, the atrophy of noradrenergic neurons in the locus coeruleus, which play a crucial role in the transition between wakefulness and sleep, is a characteristic feature of the progressive neurodegeneration observed in HD (61). Moreover, widespread cortical damage in HD contributes to SWS disturbances, as this relies on the precise synchronization of oscillatory neuronal activity throughout the brain (4). Such changes may represent a key mechanism underlying sleep architecture abnormalities in HD patients. The striatum, the primary site of neurodegeneration in HD, does not initially play a significant role in sleep regulation. However, it may contribute to sleep disturbances, primarily by generating excessive and abnormal motor activity. This mechanism is linked to disruptions in cortico-striatal interactions (4, 62, 63).

### Neuropsychiatric correlates of sleep disturbance in HD

Proper sleep is crucial for maintaining the body’s homeostasis, although its significance has yet to be fully elucidated. In healthy individuals, sleep deprivation leads to cognitive impairment, increased irritability, and impulsivity, and it may also contribute to the development of depressive disorders (64, 65). Additionally, reduced deep sleep duration has been linked to a diminished ability to form and consolidate memory traces (66). Given the well-documented negative impact of sleep disturbances on the quality of life in healthy individuals, their consequences are likely even more pronounced in patients with HD. Cognitive impairment and neuropsychiatric symptoms are central to the clinical presentation of HD, emphasizing the vital role that sleep disturbances may play in disease progression.

Studies in transgenic mouse models of HD demonstrate progressive cognitive decline closely associated with worsening sleep and circadian rhythm disturbances. Irrespective of the underlying mechanisms—whether involving aberrant input to the SCN (53), orexinergic hyperactivity (67), or reduced histaminergic and monoaminergic transmission (68)—experimental interventions that stabilized sleep and circadian patterns have consistently been associated with improved cognitive outcomes. However, findings from human studies assessing the relationship between sleep quality and cognitive function in HD mutation carriers remain inconclusive. One key challenge in interpreting these results is the variability in the methods used to evaluate sleep quality.

A 2010 study involving 84 HD mutation carriers and a control group employed a battery of sleep assessment scales. No significant differences were found between groups concerning cognitive test performance, as measured by the Unified Huntington’s Disease Rating Scale (UHDRS) cognitive subscale. However, a correlation was noted between delayed wake time and poorer cognitive test scores (15).

In a 2018 study conducted by a team of Spanish researchers, significant inverse correlations gvwere identified among a group of 38 HD mutation carriers involving sleep quality scores on the PSQI and cognitive performance on tests such as the Symbol Digit Modality Test (SDMT) (39).

Studies based solely on subjective measures, however, are not sufficiently reliable. A recent cross-sectional study compared pre-HD carriers, stratified by estimated years to diagnosis, with 42 healthy controls. It used 14-day actigraphy, sleep diaries, standardized questionnaires, and a remote cognitive battery that assessed multiple domains. The study found that higher sleep fragmentation and reduced intra-daily variability were significantly associated with poorer performance in executive function, memory, and visuospatial abilities (31). The authors emphasized that it remains unclear whether the observed sleep disturbances contribute to cognitive decline or merely reflect underlying neurodegeneration. A lack of neuroimaging data is a notable limitation in interpreting these associations.

The longitudinal study published in 2025 by Voysey et al. using polysomnography demonstrated that deficits in attention, psychomotor speed, and executive function are closely linked to sleep maintenance insomnia in individuals with HD. The data indicate that sleep continuity may have a greater impact on cognitive performance than other factors, like total sleep time. Moreover, greater insomnia severity correlated with elevated levels of NfL, a biomarker of neurodegeneration, independent of disease stage, medication use, and depressive symptoms. It is important to note that this study, like previous research, does not establish a causal relationship between sleep disturbances, cognitive decline and neurodegeneration. Its observational design hinders definitive conclusions regarding directionality. In addition, potential confounding factors—such as medication use and depressive symptoms—may influence both sleep and cognitive outcomes despite statistical adjustments. Nevertheless, the findings underscore the potential value of targeting sleep maintenance insomnia in prodromal and early-stage HD (32). Table 1 provides a summary of human investigations linking sleep and cognitive impairment in Huntington’s disease.

**Table 1:** Summary of investigations linking sleep and cognitive impairment in Huntington’s disease.

Study	Sample size	Percentage female	Disease Severity (UHDRS)	Methods	Findings in HD patients
Aziz et al. (2010) (15)	168:21 premanifest, 63 manifest, 84 HC	57.7 premanifest 54% manifest 57.1% HC	UHDRS motor: 1.9 (premanifest), 28.9 (manifest) UHDRS cognitive: 254 (premanifest), 187 (manifest)	Questionnaires: ESS, PSQI, BDI, SCOPA-SLEEP	More night-time impairment, More daytime dysfunction, Later wake-up time significantly associated with cognitive score and depressive symptoms, Depression as the only independent correlate of nighttime sleep impairment.
Baker et al. (2016) (92)	67:32 manifest (17 with sleep problems), 35 premanifest (14 with sleep problems)	61% manifest 60% premanifest	UHDRS motor: premanifest no sleep problems 1.1 ± 1.2 premanifest with sleep problems 0.6 ± 1.2 manifest without sleep problems 18.3 ± 9.5 manifest with sleep problems 17.5 ± 12.5	Questionnaires: BDI, SDMT, STROOP-WORD, HADS, Frontal Systems Behavior Scale, SCOPI Structural MRI with volumetric analysis	No significant differences in neurocognitive measures between those with and without reported sleep problems, Manifest with sleep problems had significantly accelerated thalamic degeneration and poorer neuropsychiatric outcomes compared to those without sleep problems, Premanifest with sleep problems had significantly poorer neuropsychiatric outcomes compared to those without.
Diago et al. (2018) (39)	76:23 premanifest, 9 manifest, 39 HC	65.2% premanifest 60% manifest 63.2% HC	UHDRS motor: premanifest 0.43 ± 1.08 manifest 26.6 ± 21.42; UHDRS cognitive: premanifest 309.3 ± 47.03 manifest 181.33 ± 89.43	Questionnaires: PSQI, ESS, UHDRS cognitive, HADS, Irritability Scale	Mutation carriers were significantly more depressed and had lower cognitive scores than non-carriers, HD patients had worse sleep quality associated with more severe cognitive impairment and higher anxiety, depression and irritability scores, Habitual wake-up time was delayed and associated with worse cognitive performance and greater depressive and anxiety symptoms.
Tanigaki et al. (2020) (92)	51:28 HD gene carriers, 22 caregivers	69% HD gene carriers 55% caregivers	No data	Questionnaires: PSQI, ESS, University of Cambridge HD Sleep Questionnaire, STOP questionnaire for Obstructive Sleep Apnea, HADS, NeuroQoL v2.0 Cognitive Function—Short Form	Diminished subjective sleep quality and increased sleep latency significantly associated with elevated levels of anxiety and depression, as well as with poorer self-perceived cognitive performance, Both HD gene carriers and their caregivers exhibited a high prevalence of poor sleep quality, exceeding that of the general population.
Fitzgerald et al. (2025) (64)	78:36 premanifest, 42 HC	69.4% premanifest 66.7% HC	UHDRS total functioning capacity score: 13	Questionnaires: PSQI, ESS, ISS, Fatigue Severity Scale Actigraphy Battery of standardized cognitive tests	Pre-HD with higher wake after sleep onset and greater fragmentation index performed worse on executive functions, verbal learning and memory tests, Participants with higher interdaily stability (more stable patterns) performed worse on cognitive tasks, Poorer self-reported sleep quality was related to worse performance in memory, visuospatial, and paced tapping.
Voysey et al. (2025) (32)	48:28 HD (premanifest in baseline), 28 HC	57.1% premanifest 67.9% HC	UHDRS diagnostic confidence level 0–1 at baseline	Polysomnography (baseline+12-year follow-up) Actigraphy (baseline+ 10-year follow-up) serum NfL (baseline+12-year follow-up), battery of standardized cognitive tests	Greater sleep maintenance insomnia was associated with cognitive impairment and with higher NfL levels, Sleep stage instability begins early in the premanifest phase, Sleep maintenance insomnia appears closer to phenoconversion.

HC, healthy controls; UHDRS, Unified Huntington Disease Rating Scale; ESS, Epworth’s Sleepiness Scale; PSQI, Pittsburgh Sleep Quality Index; BDI, Beck Depression Inventory; SDMT, Symbol digit Modalities Test; HADS, Hospital Anxiety and Depression Scale; SCOPI, Schedule of Obsessions, Compulsions and Psychological Impulses; MRI, Magnetic Resonance Imaging; ISS, Insomnia Severity Scale; NfL, neurofilament light.

Depression is the most common neuropsychiatric disorder in HD and has been consistently correlated with sleep disturbances across studies (14, 37, 39, 69). However, the direction of this relationship remains unclear. Intuitively, poor sleep quality may exacerbate depressive symptoms, a pattern well-documented in the healthy population. In other neurodegenerative diseases, such as Alzheimer’s and PD, effective treatment of depression has been associated with a reduction in sleep disturbances (70, 71). However, no data are currently available to confirm a similar relationship in HD.

### Therapeutic strategies

Regardless of etiology, the foundation of effective sleep disorder management lies in non-pharmacological interventions. A key component is adherence to proper sleep hygiene, which includes maintaining a consistent daily schedule with fixed wake and sleep times. Additionally, incorporating regular and varied physical activity, avoiding daytime naps, and limiting caffeine intake in the afternoon and evening are essential strategies. Implementing these changes is relatively simple and, most importantly, carries no risk of adverse effects.

While pharmacotherapy may influence sleep patterns, it is essential to emphasize that medications are not the sole contributors to sleep disturbances in HD. Alterations in sleep architecture can already be detected in the premanifest stage, before any medical treatment is initiated (21, 26, 32). The observation that sleep and circadian abnormalities in HD animal models can be both induced through targeted genetic manipulation—such as mHTT-Q128 expression in subsets of circadian pacemaker and sleep-regulating neurons in the Drosophila model—and ameliorated by interventions enhancing autophagy indicates that disturbances in sleep and circadian rhythms are an inherent feature of HD pathology (72).

Nevertheless, the systematic evaluation of medications for their impact on sleep quality and circadian rhythms is important. If discontinuing drugs that negatively affect sleep is not feasible, adjusting their administration schedule should be considered. Stimulating medications should be taken in the morning, while sedative agents are best administered in the evening.

Dopamine antagonists, such as tiapride and haloperidol, influence clock gene expression in the SCN, but their effects are highly time-dependent. The least disruption occurs when these medications are taken immediately before sleep (73). For sedative neuroleptics, olanzapine and quetiapine are recommended due to their more favorable profile in the context of sleep disturbances (74). Yet antipsychotic medications have been linked to worsening clinical outcomes in individuals with Alzheimer’s disease and other dementias, including higher risks of cognitive decline, cerebrovascular events, hip fractures, and mortality. Consequently, their use for treating sleep disturbances is discouraged unless clinically justified for coexisting behavioral or psychological symptoms (75).

Selective serotonin reuptake inhibitors (SSRIs), frequently prescribed in this patient population, can disrupt sleep and cause insomnia in approximately 10–20% of patients (76, 77). As an alternative, antidepressants with additional antihistaminergic properties, such as mirtazapine, or potent 5-HT2 receptor antagonists, such as trazodone, may be considered (65, 77). However, both of these medications have anticholinergic properties that can lead to drowsiness and cognitive impairment. Therefore, their use should be limited to patients who also exhibit depressive symptoms, rather than being prescribed solely for the treatment of sleep disturbances (75).

Although less commonly discussed, medications not primarily targeting mood or psychosis may also interfere with sleep–wake regulation in HD. A compelling case report described a 49-year-old woman with manifest HD who developed delayed sleep phase syndrome following dose-dependent exposure to memantine, an NMDA receptor antagonist sometimes used off-label to manage cognitive or motor symptoms in neurodegenerative disorders. Notably, the patient had no prior sleep complaints. Still, after titration to 20 mg of memantine, she experienced a significant 2–3-h delay in sleep onset and morning wake time, objectively confirmed by actigraphy. Reducing the dose to 10 mg reversed the circadian disruption without compromising motor symptom control (78). This case highlights how even medications considered relatively benign in other populations may have unanticipated chronobiological effects in HD.

Daily administration of alprazolam to transgenic R6/2 mice stabilized clock gene expression in the SCN and improved cognitive function (53). However, the lack of studies involving HD patients and the high addictive potential of benzodiazepines limit their use to short-term treatment (less than two weeks) in cases where other approaches have failed (65). An alternative may be MT1 and MT2 melatonin receptor agonists, which, when administered in the evening, can mimic the physiological rise in melatonin and support circadian rhythm regulation (79). Recommended medications include exogenous melatonin, agomelatine (which also exhibits antidepressant effects due to 5-HT2C receptor antagonism), ramelteon, and tasimelteon. A 2016 Cochrane review on pharmacotherapy for sleep disturbances in dementia syndromes found no clear benefits of melatonin or ramelteon but emphasized their safety and the absence of significant adverse effects (80).

Marking a significant step forward in the clinical evaluation of sleep-targeted therapies for HD, a recent randomized, double-blind, placebo-controlled crossover pilot trial directly investigated the efficacy and safety of melatonin in HD gene expansion carriers (HDGECs) experiencing sleep disturbances. Among 114 individuals screened, 15 premanifest or early-manifest HDGECs (mean age: 46.5 ± 13.9 years; 7 females, 8 males; mean CAG repeat length: 43.8 ± 2.9) met the eligibility criteria. Participants were randomly assigned into two arms: one receiving melatonin first, followed by placebo (M–P), and the other receiving placebo first, followed by melatonin (P–M). Each treatment period lasted four weeks, separated by a two-week washout interval, during which participants received 3 mg of oral melatonin nightly. Sleep-related outcomes were assessed using the PSQI (excluding the medication-use component), Huntington’s Disease Sleep Questionnaire (HD-SQ), and the ESS. Additional endpoints included cognitive assessments, neuropsychiatric symptoms, motor performance, and clinician global impression. The trial found no statistically significant differences between melatonin and placebo across all primary and secondary outcome measures. Sleep quality, daytime sleepiness, cognitive functioning, mood, and motor symptoms remained stable throughout both treatment phases. A small, non-significant increase in ESS scores was observed during melatonin treatment (6.1 vs. 5.0; *p* = 0.23). Importantly, melatonin was well tolerated, and no adverse events were reported. The lack of anticipated effect may result from certain methodological limitations. In particular, sleep outcomes were evaluated solely using subjective measures, without incorporating objective techniques. Additionally, the use of an immediate-release melatonin formulation may have limited its effectiveness in maintaining sleep. This highlights the need for future studies to use prolonged-release preparations (81). While preliminary, this study demonstrates the feasibility and safety of melatonin administration in individuals with HD and underscores the urgent need for larger, adequately powered trials to evaluate its therapeutic potential in targeting circadian dysfunction and sleep-related symptoms in this population.

Dual orexin receptor antagonists (DORAs) are a new class of medications that target the orexin signaling pathway and have shown effectiveness in treating insomnia. Clinical studies indicate that DORAs can improve sleep quality in patients with AD who experience sleep disturbances and circadian rhythm disorders (82). Additionally, preclinical research has demonstrated that acute administration of the dual orexin receptor antagonist suvorexant in the R6/1 mouse model of HD improved sleep continuity and alleviated cognitive deficits (67). Clinical trials in the human HD population are necessary to assess their efficacy and safety in this context.

Bright light therapy (BLT) is a non-pharmacological method for regulating circadian rhythms. Exposing individuals to high-intensity light at a specific time of day, enables the phase shifting of the biological clock, thereby influencing the overall sleep–wake cycle. The physiological mechanism underlying BLT involves the activation of melanopsin, a photopigment found in intrinsically photosensitive retinal ganglion cells. The resulting electrical impulses are transmitted through the retinohypothalamic pathway to the SCN, and then, via polysynaptic circuits, to the pineal gland. Light exposure inhibits melatonin production, while secretion of this hormone increases in darkness. Standard parameters for BLT include a light intensity of 10,000 lux. The lamps utilized in this therapy are fitted with UV filters to ensure safety during treatment.

BLT combined with a regular physical activity schedule has been shown to delay the disruption of circadian activity patterns in transgenic R6/2 mice (83). However, no studies have yet been conducted to assess its efficacy in HD patients. Research in other neurodegenerative diseases provides more data on its potential benefits.

Studies in small groups of PD patients (fewer than 20 participants) suggest that BLT may positively impact sleep parameters, mood, and motor symptoms (84). In 2019, a randomized trial involving 83 PD patients with comorbid depressive symptoms was conducted. A three-month intervention with 10,000-lux light therapy, compared to a control light condition (200 lux), significantly improved subjective sleep measures. However, no significant differences were observed between groups regarding depressive symptoms (85). A 2014 Cochrane review that included randomized controlled trials of light therapy across dementia types and severities found no consistent benefits for circadian regulation or sleep outcomes (86). However, a recent controlled study involving 14 dementia patients in long-term care demonstrated that a tailored lighting intervention—designed to deliver a circadian stimulus (CS) of ≥ 0.3 over two 8-week periods—led to improved actigraphy-derived sleep parameters and mood ratings compared to baseline (87). These findings highlight the therapeutic potential of tailored lighting, designed based on the CS measure, on improving sleep and mood in patients with neurodegenerative diseases. This suggests that similar approaches could be explored for managing sleep disturbances in patients with HD.

Beyond pharmacological and light-based interventions, behavioral strategies targeting circadian alignment are gaining interest in HD research. One promising approach is Time-Restricted Feeding (TRF), a dietary regimen in which food intake is confined to a defined window of the day (typically 6–10 h), aligned with the organism’s active phase. Unlike caloric restriction, TRF maintains energy intake while restructuring its temporal pattern, thereby leveraging circadian biology to restore metabolic and behavioral rhythms.

In preclinical HD models, particularly R6/2 and BACHD mice, TRF significantly improved motor function, sleep–wake rhythmicity, and metabolic outcomes. A comprehensive review of these findings emphasizes that TRF stabilizes peripheral clock gene expression, reduces neuroinflammation, and enhances mitochondrial function. Furthermore, TRF mitigated weight loss and delayed the onset of motor decline in R6/2 mice when initiated in the early stages of the disease (88). Feeding schedules may act as strong non-photic Zeitgebers, capable of entraining circadian rhythms independently of the SCN (89).

To date, no clinical trial has been published assessing the effects of TRF in individuals with Huntington’s disease. A recently published protocol outlines a 12-week interventional study designed to evaluate the feasibility, safety, and preliminary efficacy of TRF in gene-expansion carriers at an early disease stage. Participants aged 30 to 65 will adopt a consistent 10-h feeding window aligned with morning to afternoon hours. The study will also assess changes in circadian alignment markers, aiming to determine whether structured meal timing can promote circadian rhythm stabilization and improve symptom management in the HD population (90).

As previously mentioned, excessive daytime sleepiness is uncommon among HD patients (25). Its treatment primarily involves substances used in PD therapy, such as modafinil and caffeine (66). Another potential therapeutic option includes drugs that modulate the histamine H3 receptor. The H3 receptor antagonist pitolisant enhances histaminergic activity, promoting wakefulness (58). In HD patients exhibiting a phase shift in histamine secretion—characterized by increased nocturnal release and reduced daytime levels—pitolisant may represent a promising therapeutic approach (91).

Despite advances in understanding sleep and circadian rhythm disturbances in HD, systematic research on their treatment remains limited. Current therapeutic strategies are primarily based on findings from other neurodegenerative diseases and studies in animal models. However, the lack of large-scale randomized clinical trials in HD patients restricts the ability to assess the efficacy and safety of these interventions, hindering the development of standardized treatment guidelines. Future research should focus on improving sleep parameters and evaluating their impact on cognitive function and psychiatric symptoms. A better understanding of the interplay between sleep disturbances and other aspects of HD could provide more comprehensive therapeutic strategies.

## Conclusion

Sleep and circadian rhythm disturbances represent a significant yet often overlooked clinical aspect of HD. Objective assessments consistently demonstrate reduced sleep efficiency, significant sleep fragmentation, and decreased REM sleep. Emerging evidence indicates that these disturbances not only accompany the disease but may also precede its clinical onset. In premanifest individuals, difficulty maintaining sleep has been associated with cognitive impairment and elevated markers of neurodegeneration, highlighting its potential as an early biomarker.

Evidence suggests that sleep disturbances may contribute to cognitive decline and exacerbate psychiatric symptoms. Multiple preclinical studies indicate that improving sleep or circadian rhythm through pharmacological, behavioral, or light-based interventions can lead to better cognitive function, motor performance, and even prolonged survival. Despite advances in understanding the underlying pathological mechanisms, systematic research on therapeutic strategies targeting sleep and circadian abnormalities in the HD population remains limited. Current approaches primarily rely on insights derived from other neurodegenerative disorders. To date, the only randomized, double-blind, placebo-controlled trial investigated the efficacy and safety of melatonin. Still, it did not confirm its benefits in improving sleep quality, cognitive performance, or neuropsychiatric symptoms (81). A recently proposed protocol describes an interventional trial designed to assess the safety and efficacy of time-restricted feeding in the early stages of the disease, offering a promising, non-invasive, and relatively low-cost approach to mitigating circadian rhythm disturbances (90).

This growing interest in therapeutic approaches targeting sleep and circadian dysfunction in HD underscores the need for further interventional studies in larger patient cohorts, incorporating assessments of cognitive burden and biomarkers of disease activity. Understanding and treating sleep and circadian dysfunction offers a promising avenue to improve quality of life and potentially modify disease burden in HD.
